# Multiscale Contextual Mamba: Advancing Psychiatric Disorder Detection across Multisite Functional Magnetic Resonance Imaging Datasets via State Space Modeling

**DOI:** 10.34133/hds.0224

**Published:** 2025-08-05

**Authors:** Shusheng Li, Yang Bo, Yuchu Chen, Jianfeng Cao, Bo Bi, Ting Ma, Chenfei Ye

**Affiliations:** ^1^ International Research Institute for Artificial Intelligence, Harbin Institute of Technology (Shenzhen), Shenzhen, China.; ^2^ Huawei (Hong Kong), Hong Kong, China.; ^3^ Department of Electronic and Information Engineering, Harbin Institute of Technology (Shenzhen), Shenzhen, China.; ^4^ School of Biomedical Engineering, Harbin Institute of Technology (Shenzhen), Shenzhen, China.; ^5^ Department of Clinical Psychology, The Eighth Affiliated Hospital, Sun Yat-Sen University, Shenzhen, China.; ^6^ Peng Cheng Laboratory, Shenzhen, China.

## Abstract

**Background:** Major depressive disorder (MDD) and autism spectrum disorder (ASD) are complex and heterogeneous neuropsychiatric disorders with overlapping symptoms, presenting remarkable challenges for accurate diagnosis. Leveraging functional neuroimaging data offers an opportunity to develop more robust, data-driven approach for psychiatric disorder detection. However, existing methods often struggle to capture the long-term dependencies and dynamic patterns inherent in such data, particularly across diverse imaging sites. **Methods:** We propose Multiscale Contextual Mamba (MSC-Mamba), a Mamba-based model designed for capturing long-term dependencies in multivariate time-series data while maintaining linear scalability, allowing us to account for long-range interactions and subtle dynamic patterns within the brain’s functional networks. One of the main advantages of MSC-Mamba is its ability to leverage the distinct characteristics of time-series data, allowing it to generate meaningful contextual information across various scales. This method effectively addresses both channel-mixing and channel-independence scenarios, facilitating the selection of relevant features for prediction by considering both global and local contexts at multiple scales. **Results:** Two large-scale multisite functional magnetic resonance imaging datasets, including REST-meta-MDD (*n* = 1,642) and Autism Brain Imaging Data Exchange (ABIDE) (*n* = 1,022), were used to validate the performance of our proposed approach. MSC-Mamba has achieved state-of-the-art performance, with an accuracy of 69.91% for MDD detection and 73.08% for ASD detection. The results demonstrate the model’s robust generalization across imaging sites and its sensitivity to intricate brain network dynamics. **Conclusions:** This paper demonstrates the potential of state-space models in advancing psychiatric neuroimaging research. The findings suggest that such models can significantly enhance detection accuracy for MDD and ASD, pointing toward more reliable, data-driven diagnostic tools in psychiatric disorder detection.

## Introduction

Psychiatric disorders constitute a predominant source of substantial social and economic strain on healthcare systems globally, severely impairing the welfare of individuals afflicted [1]. Despite extensive research over several decades, the identification of unified or definitive biomarkers within the field of psychiatry remains elusive [2]. This uncertainty may stem from the fact that psychiatric diagnoses are predominantly anchored in clinical manifestations and signs, rather than on the underlying biological substrates. For instance, the diagnosis of major depressive disorder (MDD) is typically confirmed when patients exhibit a minimum of 5 out of 9 specific clinical symptoms, including but not limited to a depressed mood, anhedonia, and cognitive dysfunctions [3,4]. This approach results in significant clinical heterogeneity among patients who share the same diagnostic label [5]. Such heterogeneity has impeded researchers’ ability to identify reliable biomarkers through conventional case–control methodologies, which compare patients with the same diagnosis to healthy controls (HC) [6]. Most critically, this has impeded advancements in the efficacy and outcomes of treatments for psychiatric disorders [7].

Functional magnetic resonance imaging (fMRI) has proven effective in studying the neurobiological substrates of psychiatric disorders. The blood-oxygen-level-dependent (BOLD) time-series data derived from fMRI offer critical insights into the functional connectivity and the dynamic nature of brain networks. However, the analysis of this high-dimensional data presents significant challenges, particularly in terms of capturing both local and global temporal dependencies. Traditional machine learning approaches often fall short in addressing these complexities, leading to subpar classification outcomes for psychiatric disorders such as MDD and autism spectrum disorder (ASD). To overcome the variability in data across different imaging centers, the inherent noise, and the nonlinear interactions, sophisticated computational methods are imperative.

In recent years, the research on psychiatric disorder detection has increasingly focused on large-scale multisite fMRI datasets, recognizing that large sample sizes are crucial for deriving reliable brain–behavior relationship insights [8,9]. Deep learning methods provide the distinct advantage of automatically extracting features and constructing end-to-end classification models for mental disorders, particularly when dealing with large sample sizes. However, despite the advancements, there are still several technical hurdles that need to be overcome. Convolutional neural networks (CNNs) are inferior in capturing long-term dependencies and nonlocal correlations in data [10]. Meanwhile, graph convolutional neural networks (GCNs) are prone to over-smoothing as network depth increases, which can result in the loss of distinct features vital for effective classification [1]. Besides, creating an ideal graph representation of functional brain connectivity is a complex task, as it is highly susceptible to noise and can significantly impact the performance of the model [2]. Recently, the Transformer architecture has been increasingly applied to psychiatric disorder diagnosis based on resting-state fMRI (rs-fMRI) images, as demonstrated in studies by Dai et al. [11]. While Transformers have become the go-to models for various sequence-based tasks due to their ability to capture long-range dependencies using self-attention mechanisms, they suffer from significant limitations, especially in terms of computational efficiency and scalability [12]. Transformers typically exhibit quadratic time complexity relative to the sequence length, which leads to substantial computational and memory requirements, making them less practical for long-term time-series data and large datasets like those involved in fMRI studies.

In this paper, we propose Multiscale Contextual Mamba (MSC-Mamba) for the detection of MDD and ASD based on individual rs-fMRI data. By harnessing its capacity to reduce the computational complexities associated with long-term dependencies in time-series data [13], MSC-Mamba shows an advantage in refining the selection of pertinent features for predictive modeling within both global and local contexts across various scales, making it particularly apt for the analysis of fMRI time-series data. MSC-Mamba adeptly addresses both channel-mixing and channel independence scenarios, enabling dynamic transitions between capturing inter-region dependencies through channel mixing and focusing on the unique temporal dynamics of individual brain regions with channel independence. This flexibility is crucial for comprehensive fMRI time-series analysis, including optimizing feature extraction and enhancing predictive performance [14]. In our research, we utilized rs-fMRI data from 1,642 participants, comprising 848 individuals with MDD and 794 HC, sourced across 16 sites of the REST-meta-MDD consortium [15]. Additionally, we included 1,022 samples, with 502 individuals with ASD and 520 HC, collected from 17 sites [16], to validate MSC-Mamba. We expect that this method, by modeling complex, multidimensional data, holds the potential to enhance clinical diagnostics for psychiatric disorders.

## Methods

### Data acquisition and preprocessing

We evaluate MSC-Mamba using 2 largely aggregated multisite datasets.

1. The first dataset is the REST-meta-MDD database. rs-fMRI and 3-dimensional T1-weighted structural MRI data are gathered from all participants at each site. A consistent image preprocessing procedure is applied using the Data Processing Assistant for Resting-State fMRI (DPARSF) toolbox. This procedure involves correcting for slice timing, head motion, performing normalization, and removing confounding variables, as outlined in earlier research [17–25].

We start with 1,300 MDD patients and 1,128 HC from the REST-meta-MDD dataset. However, only 848 MDD patients and 794 HC are included in the final analysis. The remaining data are excluded based on several predefined criteria. Participants older than 65 years or younger than 18 years are excluded. Additionally, individuals with missing information on age, sex, or education are removed. Low-quality images with poor spatial normalization, excessive head motion, or inadequate coverage are also excluded. Furthermore, participants whose ReHo (regional homogeneity) map showed a spatial correlation of less than 0.6 with the group mean ReHo map are excluded. Sites with fewer than 10 participants in either the MDD or HC group are discarded. Data from site 25, which mainly consisted of elderly patients with geriatric depression, are also excluded. Finally, participants from site 4, which contained duplicate datasets, are removed, as well as any images with zero signals detected in the targeted atlas. As a result, 848 MDD patients and 794 HC are included in the final dataset for analysis.

Brain regions for each participant are defined based on a brain atlas. The atlases used in this study include the AAL-116 atlas [26] and the CC200 atlas [27,28], providing time-series data for 116 and 200 regions, respectively. The BOLD signal time series from voxels within each region of interest (ROI) are extracted and averaged. The functional connectivity between every pair of ROIs is assessed by calculating the Pearson correlation coefficient of their corresponding time series. To normalize the correlation estimates, Fisher’s *z*-transformation is applied, resulting in a 160 × 160 functional connectivity matrix for each individual. Demographic details of the dataset, including the total number of participants, mean age (±standard deviation), education level, Hamilton Depression Rating Scale (HAMD) scores, illness duration, and number of episodes, are provided in Tables 1 and 2.

**Table 1.** Demographic characteristics for participants included in primary analysis for major depressive disorder and healthy controls. The unit of duration is months.

	MDD (*N* = 848)				HC (*N* = 794)			
	Min	Max	Mean	SD	Min	Max	Mean	SD
Age	30–65	21.7–46.5	21.7–46.5	3.0–12.6	18–23	24–64	20.6–45.6	1.8–15.7
Education	3–9	15–21	9.7–14.2	1.5–4.5	5–12	15–23	9.9–15.9	1.6–4.8
HAMD	1–22	26–41	14.7–30.9	2.4–9.1	–	–	–	–
Duration	0.2–9	12–480	5.3–90.1	4.2–102.5	–	–	–	–
Episodes	1–1	1–10	1–2.4	0–1.9	–	–	–	–

**Table 2.** Demographic information of the REST-meta-MDD dataset

Site	Total	Number of MDD	Age (avg ± SD)	Sex (male/female)
S-01	146	73	31.88 ± 8.07	30/43
S-02	30	16	41.75 ± 11.13	7/9
S-04	41	18	31.67 ± 7.37	8/10
S-07	72	35	41.94 ± 11.56	13/22
S-08	87	39	31.87 ± 9.67	18/21
S-09	96	48	28.56 ± 8.62	22/26
S-10	71	45	32.73 ± 10.70	21/24
S-11	37	20	30.20 ± 9.03	10/10
S-13	36	20	32.65 ± 8.39	9/11
S-14	93	61	30.13 ± 6.91	19/42
S-15	67	30	46.47 ± 12.40	15/15
S-17	82	41	21.68 ± 2.97	14/27
S-19	49	18	34.94 ± 11.09	8/10
S-20	477	249	38.56 ± 11.82	83/166
S-21	144	79	34.14 ± 12.05	34/45
S-22	38	18	33.83 ± 9.55	7/11
S-23	45	22	26.23 ± 7.27	9/13
Total	1,642	848	34.39 ± 11.54	304/528

2. The second dataset used in this paper is the ABIDE-I database [29,30]. This dataset aggregates multimodal neuroimaging data, including T1-weighted structural brain images, rs-fMRI scans, and phenotypic information, from 17 different imaging sites worldwide. The diversity of imaging sites introduces variability in acquisition protocols and participant demographics, making it an ideal dataset for evaluating methods designed to handle heterogeneous multisite data. The original ABIDE-I dataset comprises 505 individuals diagnosed with ASD and 530 HC. For the purpose of this work, we perform stringent pre-processing and matching to ensure data quality and comparability. As a result, a total of 502 ASD patients and 520 gender-matched HC are included in our evaluation. This refined subset provides a balanced and robust foundation for examining brain connectivity patterns associated with ASD while addressing potential biases introduced by imbalanced samples. The inclusion of rs-fMRI data enables the exploration of functional connectivity and dynamic neural activity, which are critical for understanding the complex neural mechanisms underlying ASD. Table 3 contains key phenotypical information for ABIDE, including distribution of ASD and HC by sex and age and the Autism Diagnostic Observation Schedule (ADOS) score for ASD subjects.

**Table 3.** Summary of ASD and HC different sites

**Site**	ASD			HC	
	Age, avg (SD)	ADOS (SD)	Count	Age, avg (SD)	Count
CALTECH	27.4 (10.3)	13.1 (4.7)	M 15, F 4	28.0 (10.9)	M 14, F 4
CMU	26.4 (5.8)	13.1 (3.1)	M 11, F 3	26.8 (5.7)	M 10, F 3
KKI	10.0 (1.4)	12.5 (3.6)	M 16, F 4	10.0 (1.2)	M 20, F 8
LEUVEN	17.8 (5.0)	^a^ (^a^)	M 26, F 3	18.2 (5.1)	M 29, F 5
MAX MUN	26.1 (14.9)	9.5 (3.6)	M 21, F 3	24.6 (8.8)	M 27, F 1
NYU	14.7 (7.1)	11.4 (4.1)	M 65, F 10	15.7 (6.2)	M 74, F 26
OHSU	11.4 (2.2)	9.2 (3.3)	M 12, F 0	10.1 (1.1)	M 14, F 0
OLIN	16.5 (3.4)	14.1 (4.1)	M 16, F 3	16.7 (3.6)	M 13, F 2
PITT	19.0 (7.3)	12.4 (3.3)	M 25, F 4	18.9 (6.6)	M 23, F 4
SBL	35.0 (10.4)	9.2 (1.7)	M 15, F 0	33.7 (6.6)	M 15, F 0
SDSU	14.7 (1.8)	11.2 (4.3)	M 13, F 1	14.2 (1.9)	M 16, F 6
STANFORD	10.0 (1.6)	11.7 (3.3)	M 15, F 4	10.0 (1.6)	M 16, F 4
TRINITY	16.8 (3.2)	10.8 (2.9)	M 22, F 0	17.1 (3.8)	M 25, F 0
UCLA	13.0 (2.5)	10.9 (3.6)	M 48, F 6	13.0 (1.9)	M 38, F 6
UM	13.2 (2.4)	^a^ (^a^)	M 57, F 9	14.8 (3.6)	M 56, F 18
USM	23.5 (8.3)	13.0 (3.1)	M 46, F 0	21.3 (8.4)	M 25, F 0
YALE	12.7 (3.0)	11.0 (^a^)	M 20, F 8	12.7 (2.8)	M 20, F 8

M, male; F, female.

^a^ Missing data [29].

### MSC-Mamba architecture

**Fig. 1.**
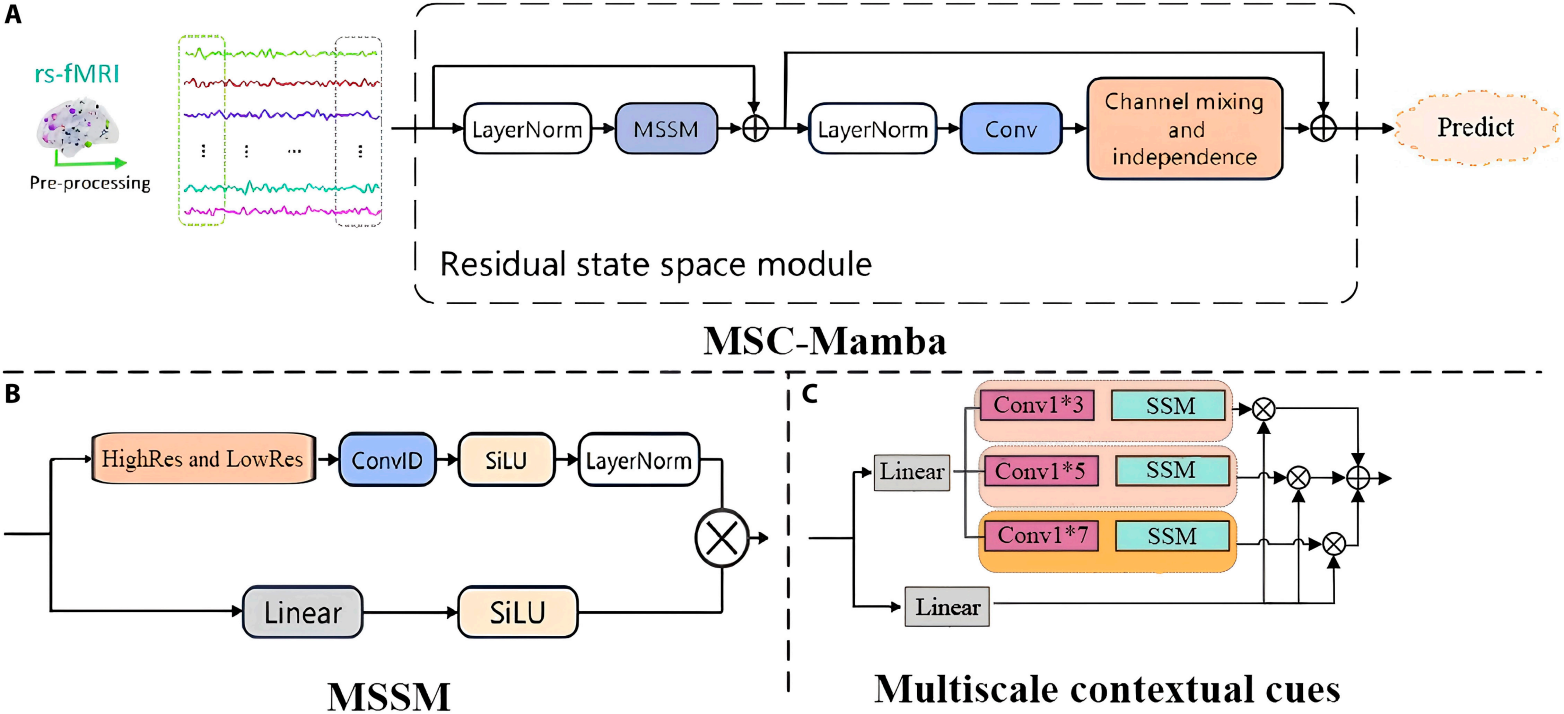
The architecture of the MSC-Mamba model, which processes resting-state fMRI data with residual state-space modeling and multiscale contextual features. (A) represents the core MSC-Mamba pipeline, where fMRI signals are preprocessed and normalized through LayerNorm, then passed into the Multiscale State Space Module (MSSM). Residual connections preserve the original information, while the output undergoes channel mixing and independence operations to enhance prediction accuracy. (B) shows the MSSM, which integrates high- and low-resolution features [obtained from (C)] through convolutions, activation functions (SiLU). (C) highlights multiscale contextual cues by convolutional filters of varying scales (e.g., 1 × 3, 1 × 5, and 1 × 7) with SSM to capture rich hierarchical information.

The architecture of MSC-Mamba is grounded in a state-space model framework, optimized for processing BOLD time-series data as shown in Fig. 1.

#### Multisite feature alignment and distribution adaptation

In multisite studies, fMRI data from different sites exhibit site-specific variations due to differences in acquisition protocols, scanner settings, and participant demographics. To ensure that the model learns consistent representations across sites while addressing these variations, we propose a class-specific feature (i.e., BOLD time-series data) alignment and distribution adaptation strategy.

To reduce site-specific biases and ensure that features are aligned across sites for the same class, we compute the alignment loss separately for each class:

$$L_{\text{align}}=\sum_{c}\sum_{s_{1},s_{2}}{\left\|F_{s_{1},c}-F_{s_{2},c}\right\|}_{2}^{2}$$ (1)

where *c* represents the class, and $F_{s_{1},c}$ and $F_{s_{2},c}$ are the features for class *c* from sites $s_{1}$ and $s_{2}$, respectively. This ensures that the features within the same class are aligned across sites, reducing the risk of misalignment between different classes.

To address the statistical discrepancies in feature distributions across sites, we apply maximum mean discrepancy (MMD) for each class separately, aligning the marginal distributions of features within the same class across different sites:

$$L_{\text{adapt}}=\sum_{c}\sum_{s_{1},s_{2}}{\text{MMD}}^{2}\left(P_{s_{1},c}\left(F\right),\;P_{s_{2},c}\left(F\right)\right)$$ (2)

where $P_{s_{1},c}\left(F\right)$ and $P_{s_{2},c}\left(F\right)$ represent the feature distributions for class *c* from sites $s_{1}$ and $s_{2}$, respectively. The empirical distribution of the features for site *s* and class *c* is represented by the following:

$$P_{s,c}\left(F\right)=\frac{1}{n}\sum_{i=1}^{n}\delta \left(f_{i}-F\right)$$ (3)

where $\delta \left(\cdot \right)$ is the discrete nature of the data. The sum is over all the feature vectors $f_{i}$ corresponding to class *c* at site *s*. These loss functions are combined in a weighted sum manner to form the total loss function:

$$L_{\text{total}}=\alpha L_{\text{align}}+\beta L_{\text{adapt}}$$ (4)

where α and β are hyperparameters that balance the contribution of each loss term. The alignment loss $L_{\text{align}}$ minimizes the feature representation differences within the same class across different imaging sites, promoting consistency and robustness of the learned features. The adaptation loss $L_{\text{adapt}}$ further aligns the feature distributions across sites, addressing statistical discrepancies and reducing site-specific biases. By performing class-specific feature alignment and distribution adaptation, we ensure that the learned representations are consistent, robust, and comparable across different sites, thus improving the model’s performance in multisite datasets.

#### State-space model in MSC-Mamba

**Fig. 2.**
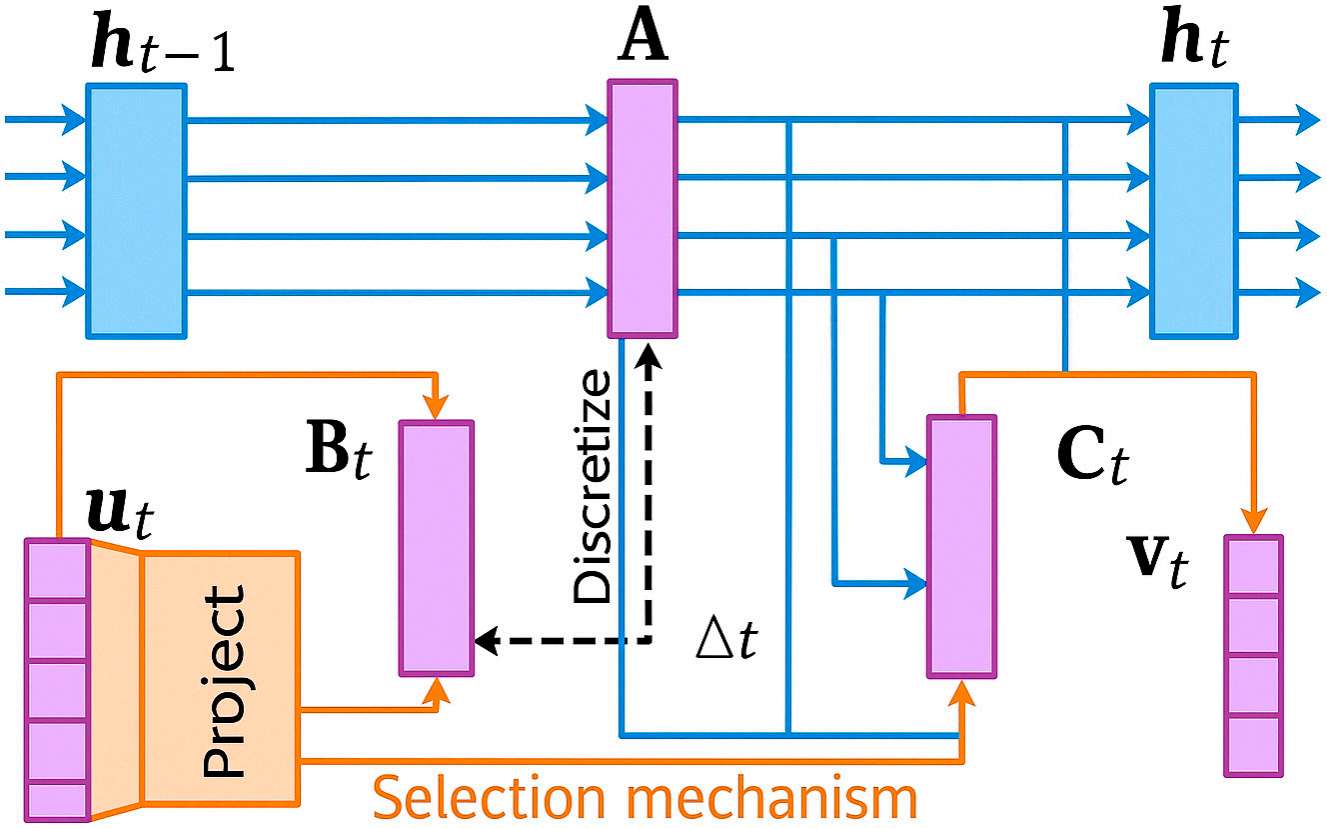
This diagram illustrates a state-space model with a selection mechanism, where the components are involved in processing and discretizing input data. The previous hidden state $h_{t-1}$ influences the current hidden state $h_{t}$ through an interaction with the attention mechanism (represented by block A), which processes the input signals. The project block ($u_{t}$) and the selection mechanism (at the bottom) interact to project and discretize the data, introducing a time delay factor Δt. The output states B*_t_*, C*_t_*, and V*_t_* represent various components of the processed data, with feedback mechanisms linking them together to form a dynamic system.

State-space models represent the state of a system as a set of variables evolving over time. These models are particularly effective for capturing temporal dependencies and dynamic behaviors in time-series data. The Mamba model integrates SSMs to model the temporal dynamics of BOLD signals efficiently, as illustrated in Fig. 2.

The continuous-time state-space model is defined as follows:

$$\frac{dh\left(t\right)}{\mathrm{dt}}=\mathrm{Ah}\left(t\right)+\mathrm{Bu}\left(t\right),v\left(t\right)=\mathrm{Ch}\left(t\right),$$ (5)

where $h\left(t\right)\in {\mathbb{R}}^{N}$ is the state vector, $u\left(t\right)\in {\mathbb{R}}^{D}$ is the input vector, $v\left(t\right)\in {\mathbb{R}}^{D}$ is the output vector, and A, B, and C are coefficient matrices that define the system dynamics. The state vector $h\left(t\right)$ captures the hidden states of the system, evolving over time based on the input vector $u\left(t\right)$. For discrete-time implementation, the continuous-time model is discretized as follows:

$$h_{k}=\bar{A}h_{k-1}+\bar{B}u_{k},v_{k}={\mathrm{Ch}}_{k},$$ (6)

where *k* denotes the discrete time steps, and $\bar{A}=\text{exp}\left(\Delta tA\right)$, $\bar{B}={\left(\Delta tA\right)}^{-1}\left(\text{exp}\left(\Delta tA\right)-I\right)B$, with Δt being the time step interval.

#### Channel mixing and independence handling

MSC-Mamba framework incorporates adaptive mechanisms for channel mixing and channel independence to address the heterogeneous nature of fMRI time-series data. These operations are dynamically integrated through a gating mechanism, enabling the model to balance inter-regional dependencies and region-specific temporal dynamics.

Channel mixing aims to capture cross-regional functional interactions by aggregating information across brain regions. Given an input feature tensor $X\in {\mathbb{R}}^{T\times C}$, where T denotes the temporal sequence length and C the number of channels (brain regions), channel mixing is implemented as a linear projection followed by nonlinear activation:

$$X_{\text{mix}}=\sigma \left(W_{\text{mix}}\cdot X+b_{\text{mix}}\right),$$ (7)

where $W_{\text{mix}}\in {\mathbb{R}}^{C\times C}$ is a learnable weight matrix that models pairwise interactions between channels, $b_{\text{mix}}$ is the bias term, and $\sigma \left(\cdot \right)$ denotes the Sigmoid Linear Unit (SiLU) activation function. This operation explicitly encodes global functional connectivity patterns, such as those observed in the default mode network or salience network, by allowing inter-channel feature interactions.

To preserve region-specific temporal characteristics, channel-independent processing is applied to each brain region individually. This is achieved through depthwise one-dimensional (1D) convolutions that operate separately on each channel:

$$X_{\text{ind}}=\text{Conv}1D\left(X;\;W_{\text{ind}}\right),$$ (8)

where $W_{\text{ind}}\in {\mathbb{R}}^{C\times K}$ represents channel-specific convolutional kernels with a temporal receptive field of size K. Unlike standard convolutions, no parameter sharing occurs across channels, ensuring that the temporal dynamics of each region (e.g., oscillatory properties of the prefrontal cortex) are modeled without interference from other regions.

A learnable gating mechanism dynamically combines channel-mixed and channel-independent features to adaptively emphasize global or local patterns based on input characteristics. The gating weights $G\in {\mathbb{R}}^{T\times C}$ are computed via sigmoid activation:

$$G=\text{Sigmoid}\left(W_{g}\cdot X+b_{g}\right),$$ (9)

where $W_{g}$ and $b_{g}$ are learnable parameters. The final output feature $X_{\text{out}}$ is a weighted combination of the 2 pathways:

$$X_{\text{out}}=G⊙X_{\text{mix}}+\left(1-G\right)⊙X_{\text{ind}},$$ (10)

where ⊙ denotes element-wise multiplication. This gating mechanism allows the model to prioritize channel mixing for regions with strong functional couplings while retaining channel independence for regions exhibiting unique temporal signatures.

#### Outer and inner Mambas

The outer Mambas operate on the high-resolution data $X^{\left(1\right)}$, capturing detailed temporal patterns. At the high-resolution level, the model processes the BOLD time-series data with minimal downsampling, retaining detailed temporal information. This level is crucial for capturing fast, transient changes in brain activity. Each outer Mamba module processes the input data through a series of linear projections, causal convolutions, and state-space transformations.

$$h_{k}^{\left(1\right)}=\bar{A}h_{k-1}^{\left(1\right)}+\bar{B}u_{k}^{\left(1\right)},v_{k}^{\left(1\right)}={\mathrm{Ch}}_{k}^{\left(1\right)},$$ (11)

where $u_{k}^{\left(1\right)}$ is the input to the outer Mamba at time step *k*, and $h_{k}^{\left(1\right)}$ is the hidden state.

The inner Mambas operate on the low-resolution data $X^{\left(2\right)}$, capturing long-term trends and dependencies. Similar to the outer Mambas, each inner Mamba module processes the input data through linear projections, causal convolutions, and state-space transformations.

$$h_{k}^{\left(2\right)}=\bar{A}h_{k-1}^{\left(2\right)}+\bar{B}u_{k}^{\left(2\right)},v_{k}^{\left(2\right)}={\mathrm{Ch}}_{k}^{\left(2\right)},$$ (12)

where $u_{k}^{\left(2\right)}$ is the input to the inner Mamba at time step *k*, and $h_{k}^{\left(2\right)}$ is the hidden state.

**Integration of Mamba outputs.** The outputs of the outer and inner Mambas are integrated to form a comprehensive representation of the input data, leveraging both high-resolution and low-resolution contexts.

$$V=\text{Concat}\left(v_{k}^{\left(1\right)},\;v_{k}^{\left(2\right)}\right),$$ (13)

where $\text{Concat}\left(\cdot \right)$ denotes the concatenation function that combines the outputs of the Mamba modules. MSC-Mamba modules are designed to capture long-term dependencies and selective attention mechanisms within the BOLD time-series data. Each module consists of the following key components:

• Linear projections. Linear transformations are applied to the input data to project it into a higher-dimensional space, facilitating the capture of complex temporal patterns.

$$u_{k}=W_{k}x_{k}+b_{k},$$ (14)

where $W_{k}$ and $b_{k}$ are learnable parameters.

• Causal convolutions. Convolutional layers with causal padding are employed to ensure that the temporal dependencies are captured without introducing future information.

$$c_{k}=\text{Conv}1D\left(u_{k}\right),$$ (15)

where $\text{Conv}1D\left(\cdot \right)$ denotes the causal convolution operation.

• Selective attention mechanisms. The selective attention mechanism in MSC-Mamba dynamically modulates temporal feature contributions based on their diagnostic relevance. This mechanism diverges from conventional self-attention by leveraging the implicit memory of state-space models, enabling linear-time computation while maintaining global temporal context. The process begins with query-key-value projections, where input features $v_{k}\in {\mathbb{R}}^{T\times D}$ are transformed via learnable matrices $W_{q},W_{k},W_{v}\in {\mathbb{R}}^{D\times d}$ into lower-dimensional representations (Q,K,V), reducing computational overhead. Crucially, instead of explicitly calculating pairwise attention scores, the SSM’s hidden state $h_{k}$ implicitly encodes temporal dependencies through its continuous-time dynamics. The attention weight $a_{k}$ at each time step k is derived by normalizing the contribution of the current state to the classification objective, formulated as $a_{k}=\text{Sigmoid}\left(\gamma \cdot ∥{\mathrm{Ch}}_{k}{∥}_{2}+\beta \right)$, where γ and β are learnable parameters, and C is the SSM’s output matrix. This approach bypasses quadratic complexity by exploiting the SSM’s sequential processing capabilities.

The attention output $a_{k}$ recalibrates features through a hybrid operation: ${\tilde{v}}_{k}=a_{k}⊙v_{k}+\left(1-a_{k}\right)⊙\text{AvgPool}\left(v_{k}\right)$, where ⊙ denotes element-wise multiplication. This design enables dual functionality—emphasizing diagnostically critical temporal segments (e.g., transient connectivity fluctuations in MDD) while suppressing noise-corrupted intervals. The mechanism inherently enhances robustness to site-specific artifacts and motion-related noise by attenuating attention to unreliable time points.

Theoretical advantages emerge from integrating attention with SSM dynamics: MSC-Mamba achieves *O*(*L*) complexity for sequence length L, outperforming Transformer-based approaches [$O\left(L^{2}\right)$] in scalability. The gating mechanism adaptively balances local detail preservation and global pattern integration across multiscale features, ensuring optimal utilization of both high-resolution transient signals and low-resolution trend information. This synergy enables precise identification of subtle psychopathology-related dynamics while maintaining computational efficiency for large-scale fMRI datasets.

• Output projections. The final output of each Mamba module is obtained through a linear projection, mapping the high-dimensional representation back to the original input space.

**Classification layer.** The integrated features are passed through a classification layer to predict the presence of MDD.

$$\hat{y}=\text{Softmax}\left(W_{k}V+b_{k}\right),$$ (16)

where $W_{k}$ and $b_{k}$ are learnable parameters, and $\text{Softmax}\left(\cdot \right)$ is the softmax activation function.

#### Training and optimization

The model is trained using a supervised learning approach with the cross-entropy loss function, which is suitable for classification tasks.

$$\begin{array}{c} L=-\frac{1}{N}\sum_{i=1}^{N}\left[y_{i}\text{log}\left({\hat{y}}_{i}\right)+\left(1-y_{i}\right)\text{log}\left(1-{\hat{y}}_{i}\right)\right], \end{array}$$ (17)

where $y_{i}$ is the true label, *N* represents the number of samples, and ${\hat{y}}_{i}$ is the predicted probability of the *i*th sample. The Adam optimizer [31] is employed for parameter updates, combining adaptive learning rates and momentum to stabilize training. The update rule is given by:

$$\theta_{t+1}=\theta_{t}-\eta \frac{{\hat{m}}_{t}}{\sqrt{{\hat{v}}_{t}}+\epsilon },$$ (18)

where θ represents the model parameters, η is the learning rate, ${\hat{m}}_{t}$ and ${\hat{v}}_{t}$ are the bias-corrected first and second moment estimates, and ϵ is a small constant to prevent division by zero.

### Experiments

Our experiments are carried out on an NVIDIA Tesla V100 with 32 GB memory. The learning rate within the network is set as 0.001, and the weight decay for regularization is 5e−4. For a fair comparison with other state-of-the-art methods on the dataset, we evaluate the performance of MSC-Mamba with different methods. Eighty percent of the data are used for model training and the remaining 20% are used as a validation set for testing. We use the SiLU [32,33] as activation functions and normalization layers (LayerNorm) [34]. The parameters of the model are fine-tuned based on the results of the validation set, allowing us to obtain the best hyperparameters. To comprehensively assess the performance of MSC-Mamba, 3 common metrics are used, including accuracy, precision, and recall.

## Results

### Methods for comparison

We evaluate MSC-Mamba against several other methods to ensure a fair comparison. These competing models are all trained using similar learning schemes, as described below. (a) 1DCNN: A conventional deep learning approach, the 1D convolutional neural network (1DCNN) has been widely used in computer vision tasks. In this experiment, we set the output channels to 128, the kernel size to 2, and the stride to 1. To prevent overfitting, batch normalization and max pooling are applied. (b) LSTM: The long short-term memory (LSTM) network is commonly employed in neuroimaging for sequence data processing. In this experiment, the LSTM has a hidden layer size of 100 and consists of a single hidden layer, with a fully connected layer added afterward for MDD classification. (c) 1DCNN_LSTM: A combination of CNN and LSTM, which are both widely used, is applied here. The CNN includes 2 layers: the first with 256 channels and the second with 64 channels. The kernel size is set to 3, and the stride is fixed at 1. The LSTM also uses a hidden layer size of 100 and one hidden layer. A fully connected layer is added at the end for classification. (d) ST-GCN: This model integrates graph convolutional networks (GCNs) with temporal convolutional networks (TCNs), as proposed by Azevedo et al. [35]. It learns spatial and temporal features from rs-fMRI data for classification tasks. (e) DKAN: The diffusion kernel attention network (DKAN) [36], proposed by Zhang et al., replaces the original dot product attention in Transformers with kernel attention to reduce parameters. Additionally, it employs a diffusion mechanism for enhanced classification performance in mental disorder prediction. (f) Transformer-Encoder model: The Transformer-Encoder model proposed by Dai et al. [11] simplifies the Transformer architecture by omitting the Decoder, reducing model complexity, and eliminating the need for complex feature selection for end-to-end classification. (g) SVM: A support vector machine (SVM) is employed as a baseline classifier, known for its robustness and effectiveness in classification tasks [37]. (h) NBS/MDMR-SVM: NBS and MDMR are state-of-the-art connectome-wide association studies (CWAS) techniques used to identify key brain network features associated with diseases. We apply a grid search to select the NBS threshold from 0.05 to 0.15, resulting in features of varying sizes for the analysis. (i) GCN: A semi-supervised GCN is used, with feature selection performed via recursive feature elimination. Each node in the network represents a subject. (j) BrainNetCNN: BrainNetCNN uses a convolutional approach to train the connectome matrix, incorporating edge-to-edge (E2E), edge-to-node (E2N), and node-to-graph (N2G) layers. The architecture includes 2 E2E layers with 32 channels, one E2N layer with 64 output features, and one N2G layer with 30 output features. A dropout rate of 0.5 is applied, following the original design. (k) BrainGNN: The brain graph neural network (BrainGNN) introduces ROI-aware graph convolutional and ROI-selection pooling layers to predict neurological biomarkers, outperforming traditional methods for fMRI analysis. (l) HGNN and DHGNN: These models utilize hypergraph neural networks to capture complex associations between brain parcellations. We follow the original structure, modifying the classifiers for graph-based classification tasks. (m) HI-GCN and TE-HI-GCN: The hierarchical graph convolutional network (HI-GCN) links graph topology with participant similarity. We discard the transfer learning component to make a fair comparison with other models. (n) MDCN: The multivariate distance-based connectome network (MDCN) [38] is a deep learning framework that combines graph neural networks with multivariate distance matrices, achieving superior results over traditional CWAS methods in analyzing brain connectomes and classifying disorders like ASD and ADHD.

Table 4 shows the performance of various methods for classifying MDD versus NC, highlighting differences in accuracy, precision, and recall. MSC-Mamba achieves the highest accuracy at 69.91%, with a precision of 70.62% and a recall of 67.96%, indicating a well-balanced performance. LSTM, known for handling sequential data, underperforms with 52.12% accuracy, 54.23% precision, and 66.75% recall, suggesting that it may not fully exploit spatial relationships in the data. The hybrid 1DCNN_LSTM model improves performance to 56.42% accuracy, 56.90% precision, and a high recall of 70.58%, effectively capturing both spatial and temporal features. The ST-GCN model, despite its advanced capability in handling spatial–temporal graphs, achieves lower results with 51.12% accuracy, possibly due to the complexity of graph structure learning or dataset-specific characteristics. The DKAN model, which introduces a kernel attention mechanism and diffusion process, achieves 52.03% accuracy, 54.24% precision, and 62.15% recall, indicating that while innovative, it may not be as effective in this classification task. The Transformer-Encoder model performs significantly better with 67.21% accuracy, 68.60% precision, and 63.96% recall, showcasing its strength in capturing long-range dependencies. Overall, MSC-Mamba stands out with its superior balance of high accuracy and precision, demonstrating robustness and reliability in classifying MDD. The slightly lower recall of MSC-Mamba may be due to the model prioritizing precision, resulting in more false negatives, which sacrifices some recall in favor of reducing false positives.

**Table 4.** Results of different methods for MDD detection. Boldface indicates best performance.

Method	Accuracy (%)	Precision (%)	Recall (%)
1DCNN	58.72	59.62	67.52
LSTM	52.12	54.23	66.75
1DCNN_LSTM	56.42	56.90	**70.58**
ST-GCN [35]	51.12	47.54	60.24
DKAN [36]	52.03	54.24	62.15
Transformer-Encoder model [11]	67.21	68.60	63.96
MSC-Mamba	**69.91**	**70.62**	67.96

Table 5 provides a detailed comparison of classification performance on the Autism Brain Imaging Data Exchange (ABIDE) dataset across multiple methods. The results demonstrate that traditional methods like SVM and MDMR-SVM show relatively lower performance, with accuracies of 65.56% and 67.43%, respectively. Graph-based models such as GCN, BrainGNN, and BrainNetCNN improve the results slightly, with BrainNetCNN achieving an accuracy of 69.73%. Among the advanced hierarchical and hybrid graph approaches, HGNN and DHGNN stand out with accuracies of 70.96% and 71.45%, respectively. MSC-Mamba achieves the highest scores across all metrics, with an accuracy of 73.08%, a precision of 72.82%, and a recall of 74.06%. This indicates a superior ability to identify both true positives (high recall) and maintain high prediction reliability (high precision). The results suggest that the proposed method effectively captures the complex brain connectivity patterns and inter-site variability inherent in the ABIDE dataset, outperforming both traditional and state-of-the-art graph-based methods. This demonstrates the robustness and applicability of MSC-Mambah in handling heterogeneous and multisite neuroimaging data.

**Table 5.** Comparisons of the classification performance for ASD detection. Boldface indicates best performance.

Method	Accuracy (%)	Precision (%)	Recall (%)
SVM	65.56	60.99	70.00
NBS-SVM [34]	63.51	53.08	72.85
MDMR-SVM [35]	67.43	62.59	72.11
GCN [35]	69.11	71.22	67.09
BrainGNN [39]	69.31	68.64	68.18
BrainNetCNN [40]	69.73	72.54	66.73
HGNN [41]	70.96	**73.25**	69.47
DHGNN [41]	71.45	71.20	71.86
HI-GCN [42]	69.31	70.62	73.00
TE-HI-GCN [43]	71.08	71.92	72.38
MDCN [44]	72.41	71.73	73.16
MSC-Mamba	**73.08**	72.82	**74.06**

### Ablation study

**Fig. 3.**
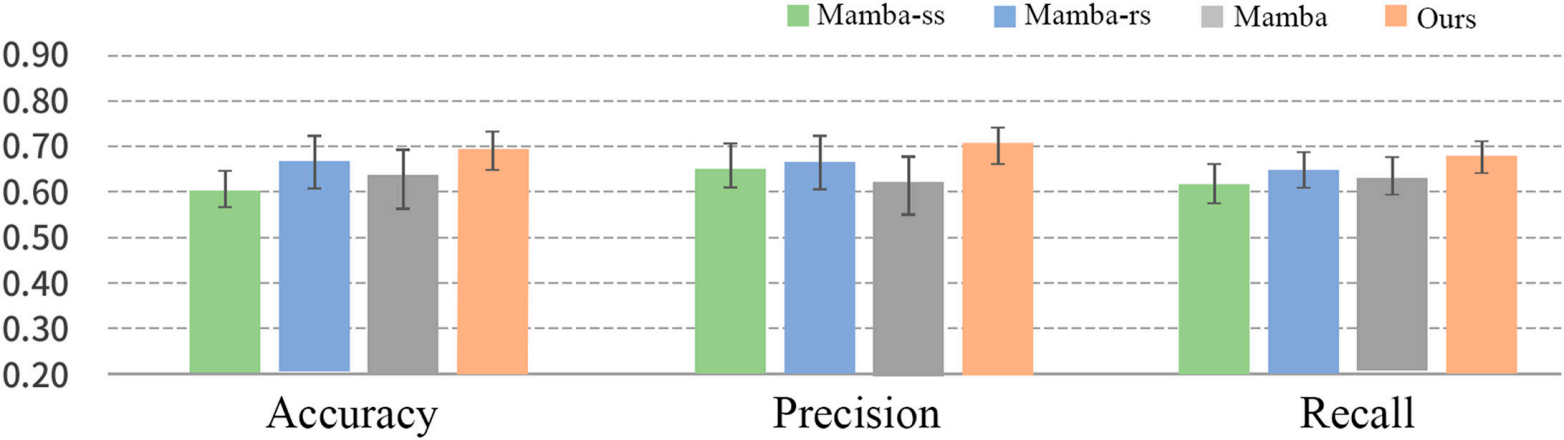
Results of the ablation study in MDD vs. HC classification.

**Fig. 4.**
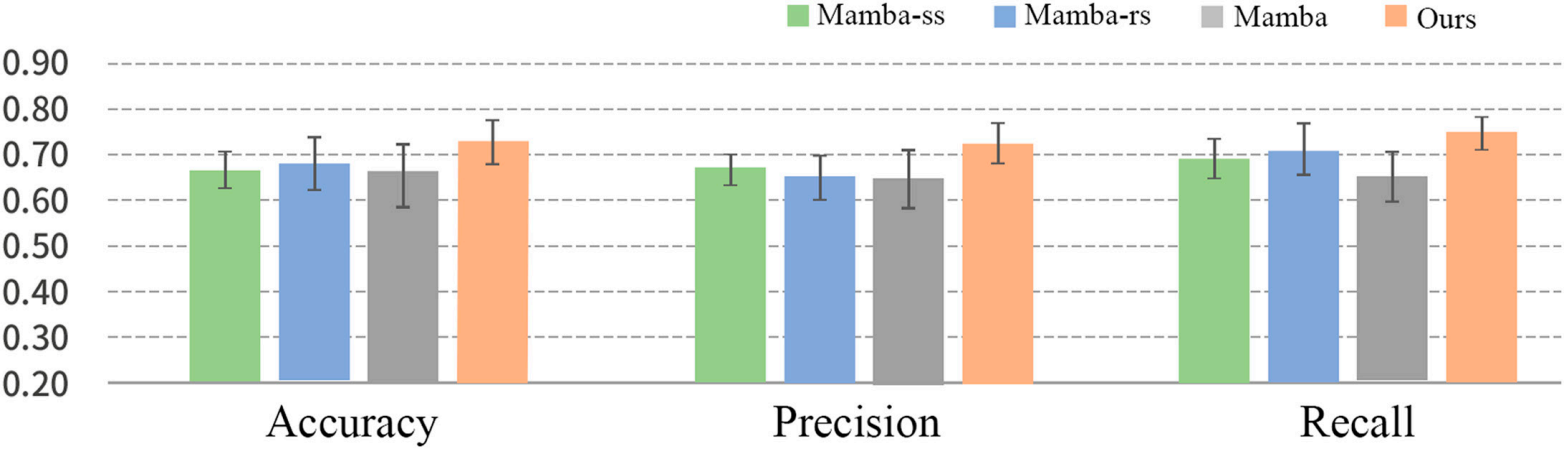
Results of the ablation study in ASD vs. HC classification.

In this section, we perform ablation experiments and verify the effectiveness of MSC-Mamba on the REST-meta-MDD dataset and ABIDE dataset as shown in Figs. 3 and 4. The ablation conditions include (a) only single-scale information (Mamba-ss) referring to the exclusive use of high-resolution data (*i.e.*, $X^{\left(1\right)}$ in the “Outer and inner Mambas” section), (b) Mamba-rs (a restricted structure without channel mixing and channel independence), and (c) the original Mamba.

The experimental results on ablation condition (a) indicate that the multiscale setting achieved the best classification performance. This success can be attributed to the model’s ability to capture features at different temporal resolutions, which is crucial for identifying patterns in complex time-series data. By using multiple convolutional layers with varying kernel sizes, the model can effectively learn both fine-grained and coarse-grained temporal features. This comprehensive feature extraction likely enhances the model’s ability to distinguish between MDD/ASD and HC more accurately. This comparison underscores the importance that fuses multiscale feature extraction with other model enhancements for optimal performance.

The experimental results of the ablation condition (b) show the effectiveness of channel mixing and channel independence. It helps stabilize training and reduce overfitting, especially for the smaller dataset. The experimental results of ablation condition (c) indicate that the original Mamba model alone does not perform well in this study. It fails to improve the model’s performance. This lack of improvement suggests that the baseline Mamba model struggles to effectively leverage the intrinsic variability of the dataset. These results underscore the limitations of the original Mamba model and the necessity of enhancements to better adapt it to the complexity of the data. From Figs. 3 and 4, we also see that MSC-Mamba (as shown in Fig. 1) with multiscale information and residual connections achieves relatively better performance in most cases. This further demonstrates the effectiveness of fusing multiscale representation with residual connections at the intermediate feature level.

### Identifying brain regions affected by MDD and ASD

**Fig. 5.**
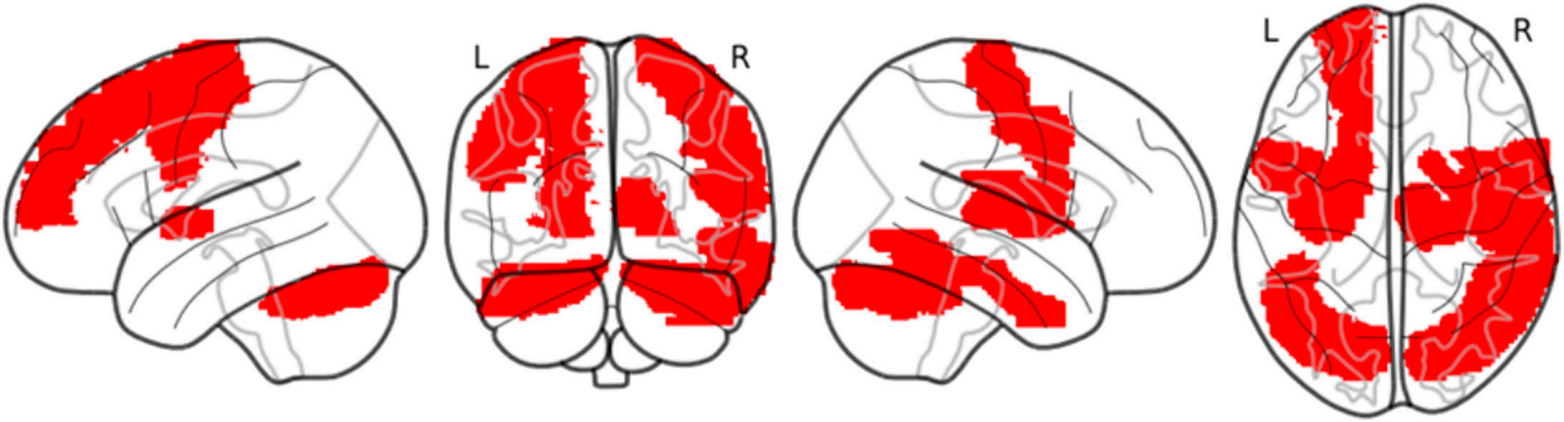
Brain regions identified using the AAL-116 atlas, highlighting areas associated with MDD.

Figure 5 illustrates related regions, visualized from multiple perspectives (left lateral, right lateral, superior, and posterior views). The red-highlighted areas correspond to specific regions of interest defined by the AAL-116 atlas, a widely used brain parcellation scheme that divides the brain into 116 anatomical regions for standardized neuroimaging analysis. We find that the highlighted regions are primarily associated with emotional regulation, cognitive control, and memory processes, which are often disrupted in MDD. These include the prefrontal cortex, anterior cingulate cortex, hippocampus, and amygdala, aligning with previous studies that emphasize the role of these regions in the pathophysiology of psychiatric disorders.

**Fig. 6.**
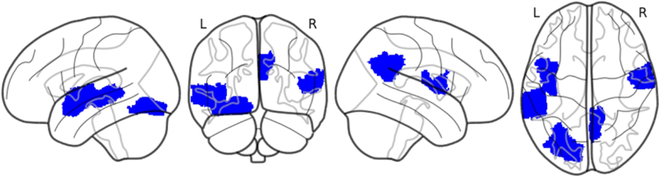
Brain regions identified using the Schaefer-100 atlas, highlighting areas associated with ASD.

Figure 6 illustrates brain regions associated with ASD based on the Schaefer-100 atlas, a parcellation scheme that divides the brain into 100 functional regions of interest. The regions highlighted in blue represent areas where significant differences have been obtained in individuals with ASD compared to neurotypical individuals. These regions are visualized from different perspectives, offering a comprehensive understanding of their spatial distribution. We find that these highlighted regions are primarily associated with social cognition, sensory processing, and repetitive behaviors. Specifically, the fusiform gyrus, superior temporal sulcus, and insula show reduced activity or connectivity, contributing to difficulties in interpreting social cues and sensory integration. Additionally, hyperconnectivity in the default mode network, including the medial prefrontal cortex and posterior cingulate cortex, suggests altered self-referential processing and reduced engagement with external stimuli, consistent with core symptoms of ASD.

## Discussion

This paper presents a novel state-space model-based framework for classifying MDD and ASD using rs-fMRI data collected from multiple imaging sites. The results demonstrate the robustness and effectiveness of MSC-Mamba in handling the complexities of neuroimaging data, including long-term temporal dependencies, high-dimensional feature spaces, and inter-site variability. Compared to traditional and state-of-the-art methods, MSC-Mamba achieves superior performance across key metrics, including accuracy, precision, and recall, for both the REST-meta-MDD and ABIDE datasets. These findings highlight the potential of the model to address critical challenges in the field of neuroimaging-based diagnostics, paving the way for its clinical and research applications.

MSC-Mamba is based on state-space models to address limitations commonly observed in traditional graph-based or deep learning methods. One key strength of this approach lies in its ability to capture long-term temporal dependencies in fMRI time-series data while maintaining computational efficiency. This contrasts with GCNs, which are limited by their focus on local neighborhood information and susceptibility to over-smoothing in deeper architectures. Similarly, while Transformer-based models have been widely adopted for sequence modeling tasks, their quadratic time complexity with respect to sequence length makes them computationally prohibitive for large-scale datasets like those used in this study. By comparison, the linear scalability of the state-space model ensures that MSC-Mamba remains efficient and applicable even for multisite datasets with high-dimensional input features.

The integration of multiscale temporal modeling within the framework further enhances its ability to capture both local and global neural dynamics. By processing data at multiple temporal resolutions, the model effectively identifies short-term fluctuations as well as long-term trends in brain activity. This multiscale approach is particularly valuable for understanding the complex patterns associated with psychiatric disorders like MDD and ASD, which often involve abnormalities at multiple levels of brain function. Additionally, the dynamic handling of channel mixing and channel independence ensures that the model can flexibly adapt to the varying characteristics of different brain regions, capturing both inter-region dependencies and region-specific temporal dynamics.

Another critical strength of MSC-Mamba is its ability to address the heterogeneity inherent in multisite neuroimaging data. Variability in imaging protocols, scanner settings, and participant demographics often introduces site-specific biases that can confound analysis and reduce model performance. MSC-Mamba mitigates these challenges through feature alignment and distribution adaptation mechanisms. By aligning feature representations across imaging sites and reducing discrepancies in both marginal and conditional data distributions, the model ensures consistent performance across diverse datasets. This adaptability makes the framework particularly well-suited for large-scale, multisite studies, where data heterogeneity is a common challenge.

Our contributions are summarized as follows: (a) Efficient long-term dependency modeling: We propose a state-of-the-art state-space model architecture that efficiently captures long-term dependencies in multivariate time-series data. This innovation addresses the inherent challenges of modeling complex temporal dynamics in fMRI data. Unlike traditional approaches such as Transformers, which suffer from quadratic time complexity and high computational demands, MSC-Mamba achieves linear scalability, making it both computationally efficient and practical for large-scale applications. (b) Enhanced classification for MDD and ASD: MSC-Mamba is based on the strengths of state-space frameworks to accurately classify MDD and ASD by capturing intricate patterns in brain activity. This approach effectively models the nuanced temporal relationships in fMRI signals, enabling robust identification of diagnostic features. The ability ensures that the model is well-suited to handling the complex temporal dynamics characteristic of psychiatric disorders. (c) Robust multisite generalization: MSC-Mamba is designed to accommodate the variability and heterogeneity present in multisite imaging data. By incorporating mechanisms to address differences in acquisition protocols, scanning conditions, and participant populations, MSC-Mamba ensures consistent and reliable performance across diverse imaging sites. This adaptability not only enhances the model’s generalizability but also supports its potential for real-world clinical deployment in large-scale studies that require integration of data from multiple sources.

Despite its promising results, the study has limitations that warrant further investigation. First, while the model demonstrates robust performance for MDD and ASD classification, its applicability to other psychiatric or neurological disorders remains unexplored. Expanding the model to additional datasets and conditions could provide further insights into its generalizability and utility. Second, while the framework incorporates feature alignment and distribution adaptation mechanisms, inter-site variability in imaging protocols may still introduce subtle biases. Future work could focus on refining these mechanisms, potentially investigating more advanced techniques such as domain-invariant feature learning or adversarial alignment.

## Conclusion

This paper presents MSC-Mamba, a state-space model-based framework designed to improve the classification of psychiatric disorders using neuroimaging data, specifically rs-fMRI. The primary challenge in neuroimaging-based psychiatric disorder classification is dealing with the inherent complexity of high-dimensional fMRI data, including long-term temporal dependencies, intersite variability, and noise across multiple imaging centers. MSC-Mamba addresses these challenges by incorporating a multiscale contextual approach, enabling the model to capture both short-term and long-term dynamic patterns in the brain’s functional networks. Additionally, the model adapts to the diverse characteristics of data collected from different sites, effectively mitigating site-specific biases that are commonly observed in large-scale multisite neuroimaging studies. The results of this study demonstrate that MSC-Mamba is capable of achieving state-of-the-art performance in the classification of both MDD and ASD across large and heterogeneous datasets. The model’s flexibility in handling multiscale features and its ability to integrate both global and local brain connectivity patterns contribute to its robustness and accuracy in detecting psychiatric conditions. Furthermore, the performance of MSC-Mamba indicates its potential for clinical applications, where reliable, data-driven diagnostic tools are essential for improving the accuracy and efficiency of psychiatric disorder detection.

## Ethical Approval

The study was conducted in accordance with ethical guidelines and received approval. All participants provided informed consent prior to their involvement in the study, ensuring that the research complied with ethical standards for human subjects.

## Data Availability

The data used in this study are publicly available from the relevant sources. The REST-meta-MDD and ABIDE datasets can be accessed via their respective platforms for further analysis and research. Detailed instructions on data access and usage policies are available on the official websites of the datasets. Researchers interested in using these datasets should comply with the terms and conditions set forth by the data providers.
